# Predicting the Vulnerability of Great Apes to Disease: The Role of Superspreaders and Their Potential Vaccination

**DOI:** 10.1371/journal.pone.0084642

**Published:** 2013-12-27

**Authors:** Charlotte Carne, Stuart Semple, Helen Morrogh-Bernard, Klaus Zuberbühler, Julia Lehmann

**Affiliations:** 1 Centre for Research in Evolutionary and Environmental Anthropology, University of Roehampton, London, United Kingdom; 2 The Orang-utan Tropical Peatland Project (OuTrop), Centre for International Cooperation in Sustainable Management of Tropical Peatland (CIMTROP), Universitas Palangka Raya, Palangka Raya, Central Kalimantan, Indonesia; 3 Exeter University, Centre for Research in Animal Behaviour, College of Life and Environmental Sciences, University of Exeter, Washington Singer Labs, Perry Road, Exeter, United Kingdom; 4 School of Psychology & Neuroscience, University of St Andrews, St Mary’s Quad, St Andrews, Fife, United Kingdom; 5 Cognitive Science Centre, University of Neuchâtel, Neuchâtel, Switzerland; Colorado State University, United States of America

## Abstract

Disease is a major concern for the conservation of great apes, and one that is likely to become increasingly relevant as deforestation and the rise of ecotourism bring humans and apes into ever closer proximity. Consequently, it is imperative that preventative measures are explored to ensure that future epidemics do not wipe out the remaining populations of these animals. In this paper, social network analysis was used to investigate vulnerability to disease in a population of wild orang-utans and a community of wild chimpanzees. Potential ‘superspreaders’ of disease - individuals with disproportionately central positions in the community or population - were identified, and the efficacy of vaccinating these individuals assessed using simulations. Three resident female orang-utans were identified as potential superspreaders, and females and unflanged males were predicted to be more influential in disease spread than flanged males. By contrast, no superspreaders were identified in the chimpanzee network, although males were significantly more central than females. In both species, simulating the vaccination of the most central individuals in the network caused a greater reduction in potential disease pathways than removing random individuals, but this effect was considerably more pronounced for orang-utans. This suggests that targeted vaccinations would have a greater impact on reducing disease spread among orang-utans than chimpanzees. Overall, these results have important implications for orang-utan and chimpanzee conservation and highlight the role that certain individuals may play in the spread of disease and its prevention by vaccination.

## Introduction

Disease is a major threat to the survival of the great apes. The emergence of Ebola and its impact on chimpanzee (*Pan troglodytes*) and gorilla (*Gorilla gorilla*) populations in western Africa has provided a clear warning of the susceptibility of great ape populations to disease [1]–[3]. Infectious diseases are now emerging at an accelerated rate in both human and animal populations [4]. The increased deforestation and forest fragmentation that is expected to occur in the future, combined with the rise of ecotourism, will increase contacts between humans and wildlife and lead to a much higher risk of inter-specific disease transmission [5]. This will be particularly problematic for the great apes, as their close phylogenetic relationship with humans means that they are likely to be susceptible to many of the same infectious diseases [6]. The slow life histories that characterise the great apes also make them particularly vulnerable to population declines, as it takes many years for populations to recover [7]–[9].

Awareness of the threat of disease to the great apes has increased considerably in recent years and guidelines relating to both visitor hygiene and behaviour have been outlined and implemented at ecotourism and research sites to prevent disease transmission from humans [10], [11]. However, these measures are often difficult to enforce, particularly among tourists who have paid considerable fees to visit the apes [12], [13], and even if all risk of disease transmission from humans was eliminated, apes would still be at risk from diseases spread from their own and other species. It is vital for the conservation of great apes that the threat of disease transmission be assessed and potential preventative measures investigated, as when epidemics occur conservationists need to be able to react quickly and in the most effective manner.

Social contacts provide the opportunity for many infectious diseases to spread within a population, and so insights into potential disease spread can be obtained using social network analysis. A social network is a graphical depiction of a social group in which individuals are represented by nodes and if two individuals have been observed to associate, their respective nodes are connected by an edge [14]. The social network approach provides a means of both visualising and analysing the way in which dyadic interactions connect individuals into an overall network, and hence the possible disease pathways within a population [15]. A wide range of species has been shown to have heterogeneous networks, indicating considerable variation in the role that individuals play in their societies [16]–[23]. This heterogeneity is also indicative of individual variation in both the probability of acquiring infection and the ability to spread infection within the group [24], [25]; for example, individuals with a lot of strong contacts or those occupying particularly central positions in the network may act as so called ‘superspreaders’, playing a disproportionately important role in disease spread [26].

Identifying potential superspreaders is important for conservation measures aimed at limiting the spread of epidemics, as these individuals could be targeted in vaccination programmes [27]. Wildlife vaccination projects have achieved a number of successes in eliminating disease to date; for example, red fox (*Vulpes vulpes*), raccoon (*Procyon lotor*) and coyote (*Canis latrans*) rabies vaccination programmes have been relatively successful so far in North America and Europe [28], and wild mountain gorillas (*G. g. beringei*) were successfully vaccinated against measles [29]. The vaccination of wild animals has disadvantages, however, as it is extremely expensive and difficult to implement, as well as being disruptive and stressful for the animals in question. Live vaccines may induce disease in the intended or even unintended hosts [30], while handling and restraining animals can cause stress, which may lower their immune response [31]. Vaccinations may lead to the selection and spread of non-vaccinal strains of the disease or reduce the selection pressure for natural resistance to diseases, although this is less likely to be a problem for highly virulent diseases for which there is usually limited natural immunity [30]. The overall drawbacks associated with vaccinations would be reduced if vaccinations were targeted at a few key individuals, or at one sex, and if doing so was sufficient to prevent a widespread epidemic [27]. This could reduce costs and effort as well as involving fewer animals in the invasive procedures. Targeting particular individuals for vaccination has not yet been widely applied; however, there is some evidence indicating that vaccinating packs of Ethiopian wolves (*Canis simensis*) that ranged within or near a corridor connecting two subpopulations reduced the overall extent of a rabies epidemic in this species [32]. It is possible that targeting superspreaders for vaccination could provide a powerful method of disease prevention, or at least limit disease spread, in wild animal populations.

The identification of superspreaders in great ape societies would provide information for conservation actions aiming to prevent large-scale disease outbreaks in these iconic species. However, it is important not only to identify such individuals but also to assess the efficacy of vaccinating these animals in comparison to simple (and potentially cheaper) random vaccinations. This assessment can be achieved by simulating the removal of individuals from their social network and measuring subsequent network fragmentation. Removal simulations can be interpreted as simulating vaccinations; removing an individual from the network removes all of the disease pathways on which it lies and effectively removes it from the disease transmission network [15], as vaccination would prevent an individual from becoming infected and would thereby prevent or at least considerably reduce the amount or duration of pathogen shedding [33]. The relative impact of vaccinating superspreaders on disease flow can therefore be assessed by simulating their removal from the network and comparing the effects of this to the removal of random individuals [16]. If a network becomes more fragmented following the removal of superspreaders than of random animals, this suggests that vaccinating the targeted animals may be an effective method of limiting disease spread in the future, as the number of disease pathways connecting individuals is reduced. Removal simulations can also be viewed as simulating death, and so in addition to telling us about the potential effects of vaccinations, they provide insights into the possible impact that the death of key individuals has on the social network. If the network becomes very fragmented following the targeted removal of central individuals, this suggests that the structure of the social system may collapse following the death of these animals [16].

The aims of this study were (i) to determine if potential superspreaders exist in two great ape species, Bornean orang-utans (*Pongo pygmaeus wurmbii*) and chimpanzees (*P. t. schweinfurthii*), and (ii) to model how vaccinating highly central individuals affects predicted susceptibility to the spread of disease in these two species. This study focussed on 46 independent orang-utans from a population in the Sabangau peat-swamp forest in Central Kalimantan, Indonesia, and 55 members of the Sonso chimpanzee community of Budongo Forest, Uganda. Orang-utans are characterised by an individual-based fission-fusion social organisation [34]. Despite spending the majority of their time alone [35]–[37], there is evidence that orang-utans do have preferential partners and individualised relationships with others [38], [39]. The social organisation of chimpanzees is also classed as fission-fusion, but chimpanzees are considerably more gregarious than orang-utans [34], [40]. It has been suggested that the lower mortality observed in orang-utans, compared to chimpanzees and other African apes, is the result of their lower levels of gregariousness, leading to reduced disease spread [41]. Analysing the social networks of a population of orang-utans and a community of chimpanzees may thus highlight the way in which differences in gregariousness impact on disease dynamics and provide insights into the level of threat that disease poses to each species.

## Materials and Methods

### Ethics Statement

Permits and ethical approval for the field studies were obtained from the Indonesian Institute of Sciences and the Ministry of Research and Technology and the Uganda National Council for Science and Technology, the Ugandan Wildlife Authority and the National Forestry Authority.

### Study Site and Data Collection

The orang-utan data were collected from 2003–2011 as part of the OuTrop multi-disciplinary research project in collaboration with CIMTROP. The field site is located in the Natural Laboratory for the Study of Peat Swamp Forests (2°19′S 114°00′E). Data collection took place in a 9 km^2^ area of mixed-swamp forest [42]. A total of 46 independent orang-utans were observed during focal follows: four adolescent females, 10 adult females, two adolescent males, 16 unflanged males and 14 flanged males. Once a focal animal was located it would be followed for as long as 10 consecutive days or until lost. Association data (i.e. presence in the same party) were recorded for each focal individual using instantaneous sampling every five minutes. A party was defined as two or more independent individuals within 50 metres (or line of sight) of each other [42].

Chimpanzee data were collected between August 2007 and July 2010. 55 independent members of the Sonso community of Budongo forest were observed during focal follows: 12 adolescent females, 24 adult females, eight adolescent males and 11 adult males. Chimpanzee infants were defined as independent after the end of their fourth year [43]. A focal animal was followed and party composition (i.e. all individuals within 50 metres of the focal animal) was recorded in scan samples every 15 minutes.

### Association Data

The orang-utan data consisted of a total of 165,717 focal scans, recorded over a nine-year period. Nine of the 46 orang-utans (two adult females, four unflanged males and three flanged males) were never observed to associate with another identifiable orang-utan. As the analyses here focus on association patterns, these individuals were excluded from further analysis. Furthermore, these individuals will evidently have negligible or no impact on the spread of contagious disease between individuals, and would not be targeted for vaccination. For completeness, however, we ran all analyses with and without these nine individuals included, and the results were essentially the same. For brevity, therefore, we present only the results where they were not included. The chimpanzee data consisted of 34,143 focal scans recorded over a three-year period. Data were compiled over this long time period to ensure that the overall structure of the community or population was meaningfully represented.

The association data for both species were then used to construct weighted association networks, using Dyadic Association Indices (DAIs) as edge weights. These indices standardise the time observed in association in relation to observation effort [44]. In this study, this was particularly important as there was considerable variation in observation time between the 37 orang-utans, ranging from a minimum of 26 scans to a maximum of 37,345 scans. Observation time for the chimpanzee data ranged from 81 to 12,387 scans. The Dyadic Association Indices were calculated using the following equation:
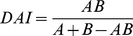
Where A is the total time that A was observed, either alone or with other independent individuals, B is the total time B was observed and AB is the total time that A and B were observed together [45]. Association indices range from zero to one, with zero indicating that two individuals were never observed together and one indicating that they were always observed together [44].

### Network Analysis

Central individuals were defined as those with high network strength, high weighted betweenness centrality or high weighted eigenvector centrality. Strength is the total weight of the edges attached to a node, so individuals with high strength are likely to have many strong relationships [14]. Weighted betweenness is measured as the number of weighted shortest paths between individuals on which a node lies; individuals with high weighted betweenness often connect individuals or groups of individuals that would not otherwise be connected [46]. Weighted eigenvector centrality incorporates both the strength of connections held by a node and the strength of connections held by the node’s neighbours. An individual with high weighted eigenvector centrality is strongly connected to a lot of nodes who also have a lot of strong connections [47]. These three measures of centrality were calculated and plotted for each individual to identify potential superspreaders, i.e. individuals with considerably higher than average values within their networks. To test whether there was a difference in centrality between the different age-sex classes in orang-utans (unflanged males, flanged males and females) node-level ANOVAs were performed in UCINET [48]. Node-level t-tests were then used to determine which classes differed significantly. As three tests were performed a Bonferroni correction was applied and relationships only viewed as significant if P<0.017. For chimpanzees, node-level t-tests were used to test for differences in centrality between male and females.

The importance of central individuals to network structure was then investigated by performing targeted and random removal simulations. To simulate the effect of vaccination or death of the most connected animals in each network, the 10 individuals with the highest strength were removed in a stepwise fashion by removing the individual with the highest strength first [49]. Following each removal, four network properties indicative of fragmentation were calculated: weighted mean shortest path length, the size of the largest cluster, the mean size of isolated clusters and the number of isolated clusters. The mean shortest path length is a measure of the average number of links needed to connect two individuals in the network and is therefore a good measure of the connectivity of a network, and consequently the speed of infectious disease spread [14]. Weighted mean shortest path length incorporates edge weight by allocating a cost to each edge based on its associated weight; edges with high association indices are given a low cost and those with low association indices a high cost. This is achieved by simply inverting the edge weight. The weighted mean shortest path length is then calculated using Dijkstra’s algorithm [50], [51]. This was normalised by multiplying by the average weight in the complete network, so that each unit represented one step of average edge weight in the complete network [52]. The size of the largest cluster, the mean size of isolated clusters and the number of isolated clusters are measures of the extent to which the network has fragmented [16], [53]. This process was then repeated, removing individuals with high weighted betweenness and then individuals with high weighted eigenvector centrality. Although individuals that have a high score on one measure of centrality are also likely to have high scores on the other measures, there will be some differences between the three sets of analyses, particularly in the order of removals. Even small differences in the identities of the removed individuals may have important consequences for network fragmentation.

The results of the targeted removals were then compared with those produced following random removals in which 10 individuals were selected at random and removed sequentially from the network. The random removal of 10 individuals was repeated 10,000 times. Targeted and random removals were performed on both the orang-utan and the chimpanzee association networks. All of the analyses were performed using igraph [54] and tnet [46] in R [55].

## Results

### Orang-utans

#### Identification of potential superspreaders

**Figure 1:**
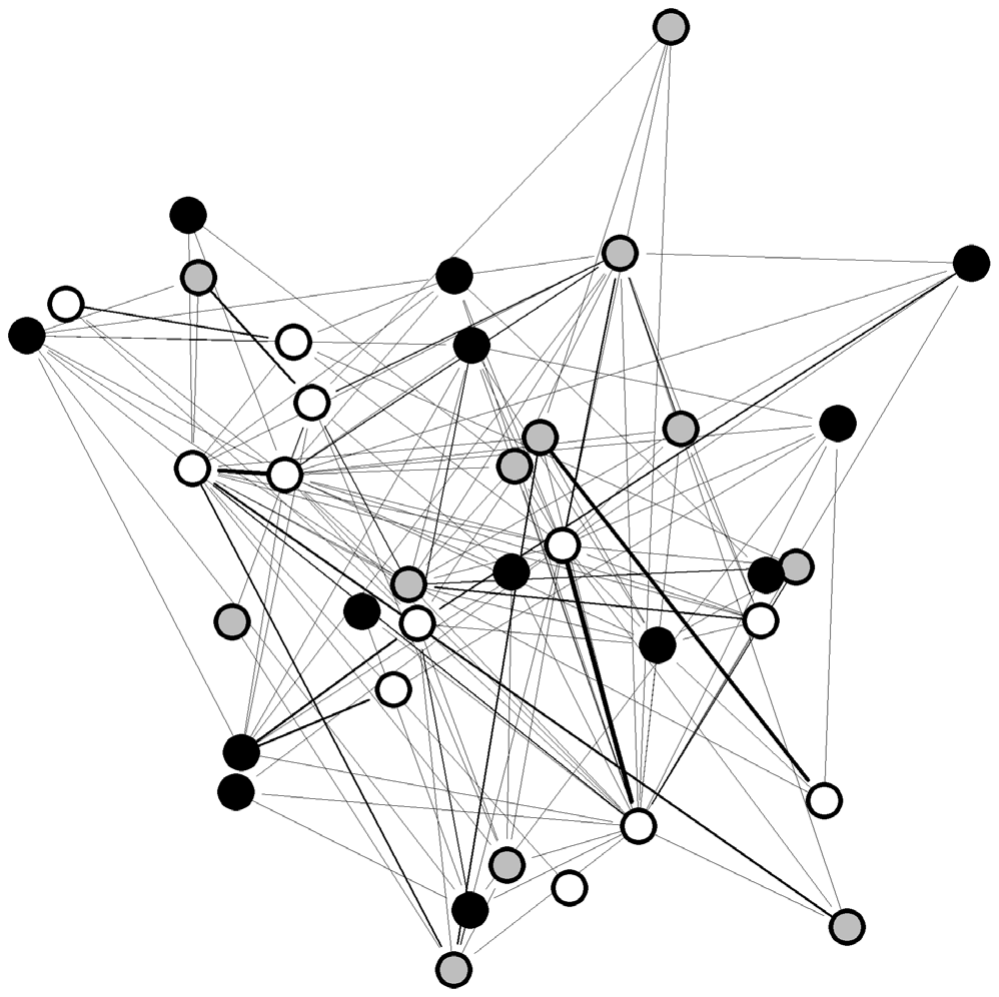
Spring-embedded sociogram of the orang-utan association network. White circles are females, grey circles are unflanged males and black circles are flanged males. Edge thickness represents the strength of the relationship.

**Figure 2:**
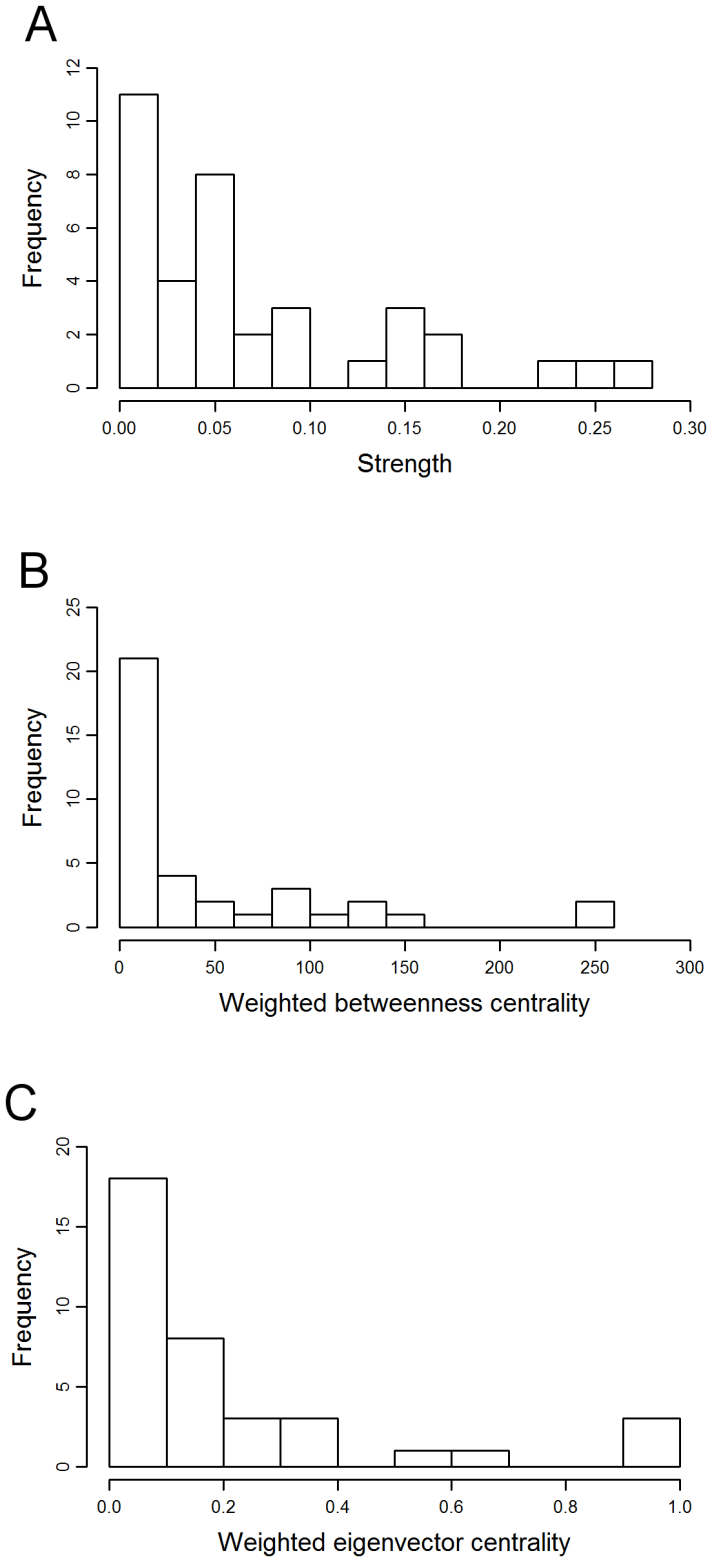
Distribution of (a) strength, (b) weighted betweenness centrality and (c) weighted eigenvector centrality values in the orang-utan network.

**Figure 3:**
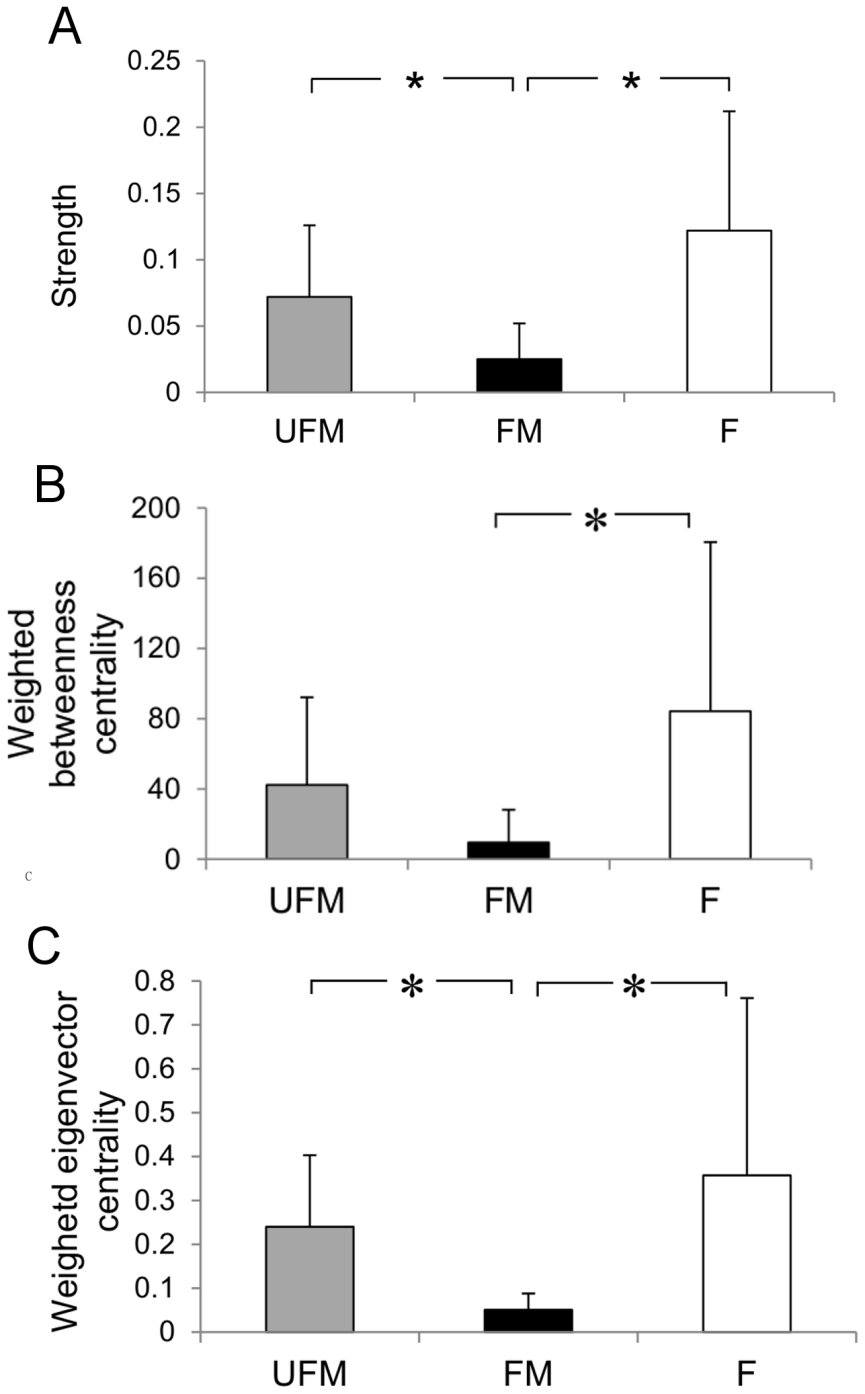
Mean (+SD) of (a) strength, (b) weighted betweenness centrality and (c) weighted eigenvector centrality scores for unflanged male (UFM), flanged male (FM) and female (F) orang-utans. Asterisks indicate significant differences after the Bonferroni correction.

The 37 orang-utans were connected by 141 edges out of a possible 666 (21%), thus the network was relatively sparse, in that most of the possible connections between individuals did not exist (Figure 1). Individuals had an average of 7.6 contacts with an average strength of 0.072. The distribution of values for strength, weighted betweenness and weighted eigenvector centrality were highly skewed; three individuals – all of them resident females - had much higher centrality than the other individuals (Figure 2). There was a significant effect of age-sex class on strength, weighted betweenness and weighted eigenvector centrality (node-level ANOVAs for strength: F = 7.682, P = 0.002; weighted betweenness: F = 4.438, P = 0.017; weighted eigenvector centrality: F = 4.834, P = 0.013). Overall, females had significantly higher centrality than flanged males, but did not differ significantly from unflanged males, while unflanged males had significantly higher strength and weighted eigenvector centrality than flanged males but did not differ significantly in weighted betweenness centrality once the Bonferroni correction had been applied (node-level t-tests for strength: females and flanged males, P<0.001; unflanged males and flanged males, P = 0.012; unflanged males and females, P = 0.114; Figure 3a; weighted betweenness centrality: females and flanged males, P = 0.008; unflanged males and flanged males, P = 0.038; unflanged males and females, P = 0.195; Figure 3b; weighted eigenvector centrality: females and flanged males, P = 0.004; unflanged males and flanged males, P<0.001; unflanged males and females, P = 0.369; Figure 3c).

#### Effect of removals

**Figure 4:**
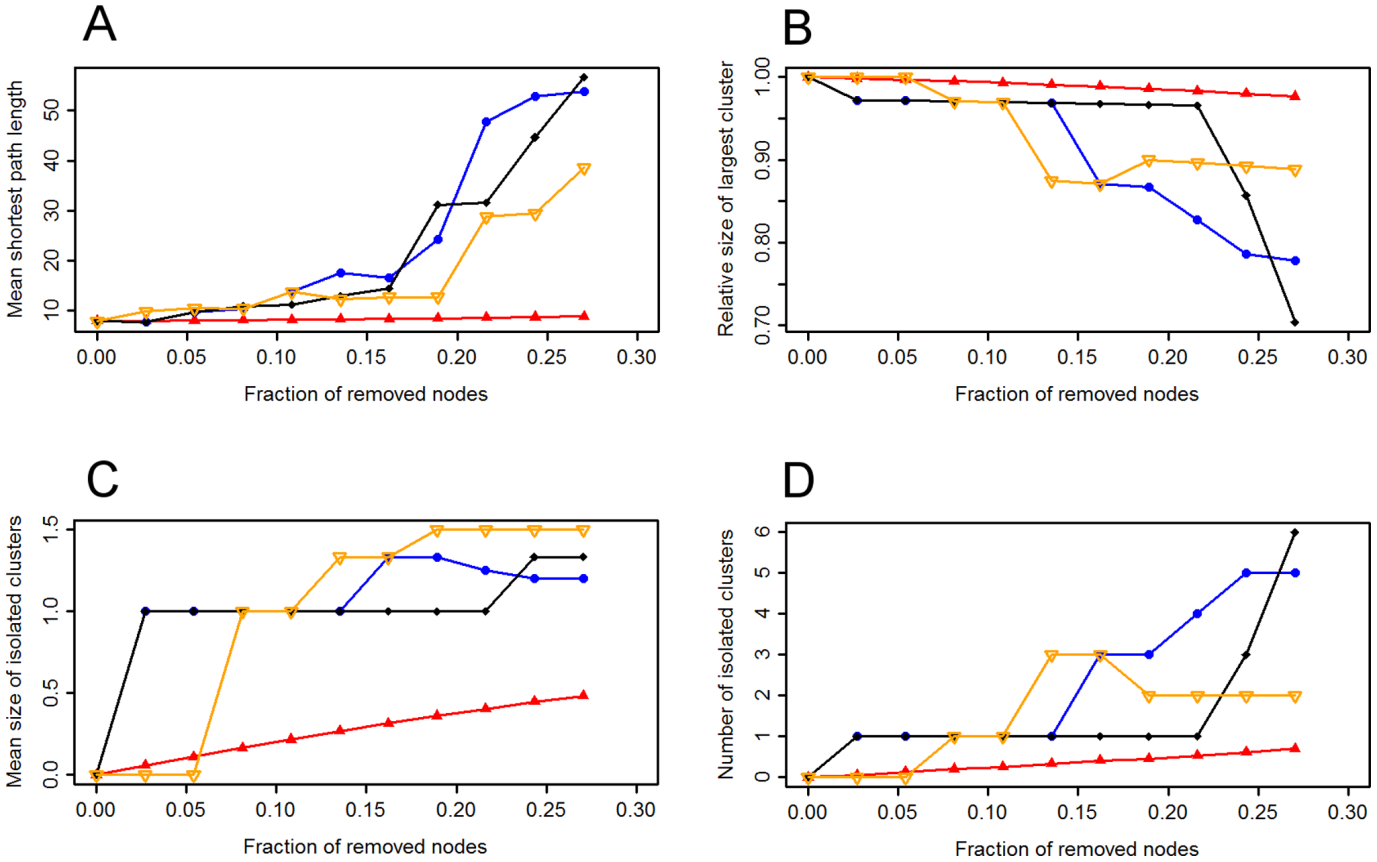
Network metrics plotted against the fraction of removed nodes in the orang-utan network. The impact of random and targeted removals on (a) the weighted mean shortest path length, (b) the size of the largest cluster (relative to the number of individuals remaining), (c) the mean size of isolated clusters and (d) the number of isolated clusters. Red triangles represent the mean of 10,000 random removals, blue squares targeted removals of individuals with the highest strength, black diamonds individuals with the highest weighted betweenness and yellow inverted triangles individuals with the highest weighted eigenvector centrality.

The orang-utan network was more vulnerable to the targeted removal of individuals with high strength, weighted betweenness and weighted eigenvector centrality than to the removal of random individuals (Figure 4). The weighted mean shortest path length under targeted removals increased much faster than under random removals. The network also became more fragmented after targeted removals compared to random removals due to a decrease in the size of the largest cluster and an increase in the mean size and number of isolated clusters. By contrast, the network appeared to be highly resilient to the removal of random individuals; the weighted mean shortest path length, the size of the largest cluster, the mean size of isolated clusters and the number of isolated clusters changed very slowly following the removal of random nodes. Most of the individuals remained in one cohesive component even after the removal of 10 random individuals (over 25% of the network).

### Chimpanzees

#### Identification of potential superspreaders

**Figure 5:**
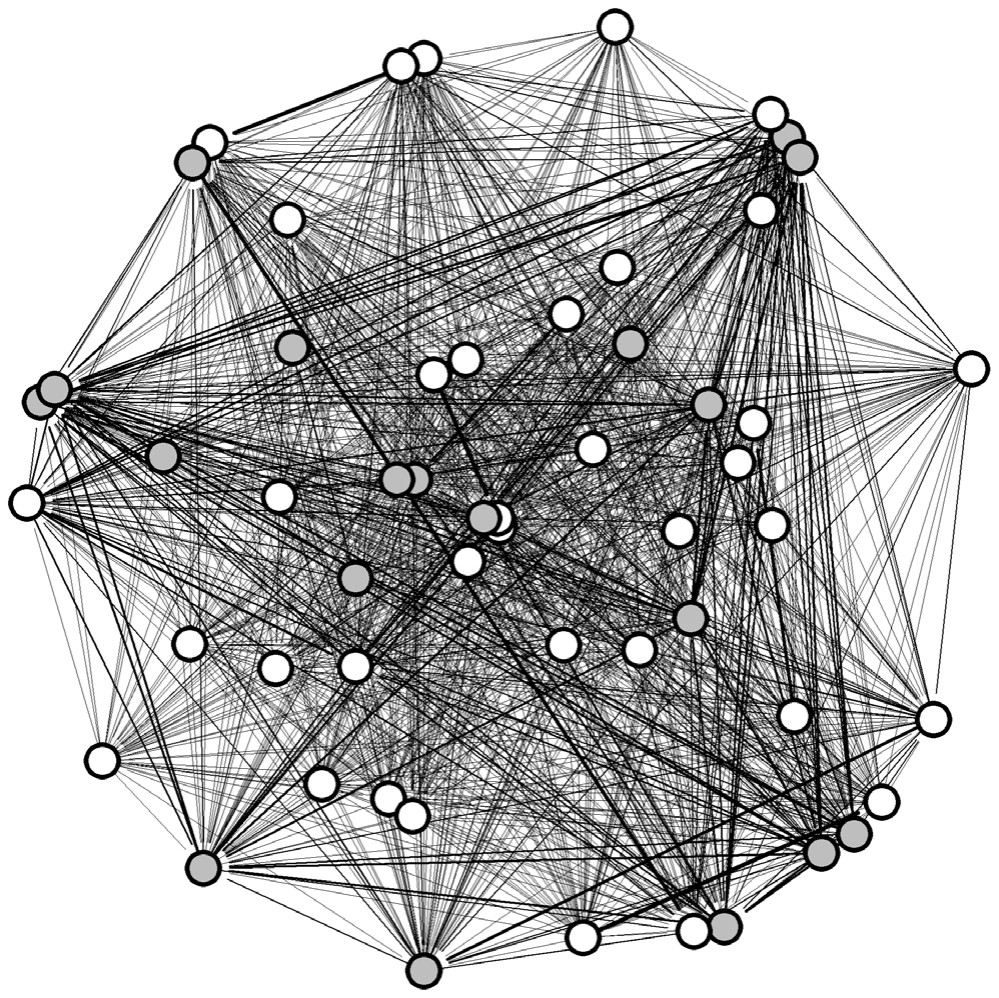
Spring-embedded sociogram of the chimpanzee association network. White circles are females and grey circles are males. Edge thickness represents the strength of the relationship.

**Figure 6:**
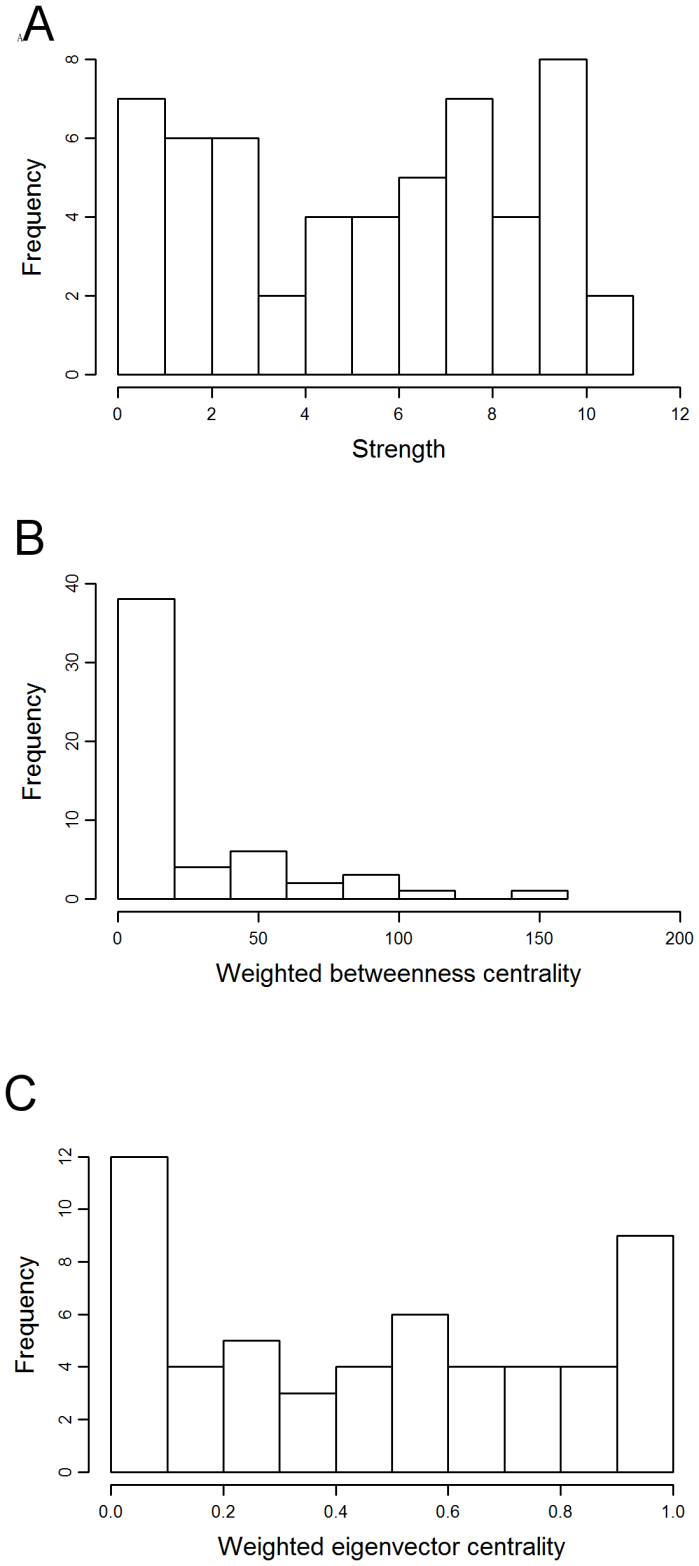
Distribution of (a) strength, (b) weighted betweenness centrality and (c) weighted eigenvector centrality values in the chimpanzee network.

**Figure 7:**
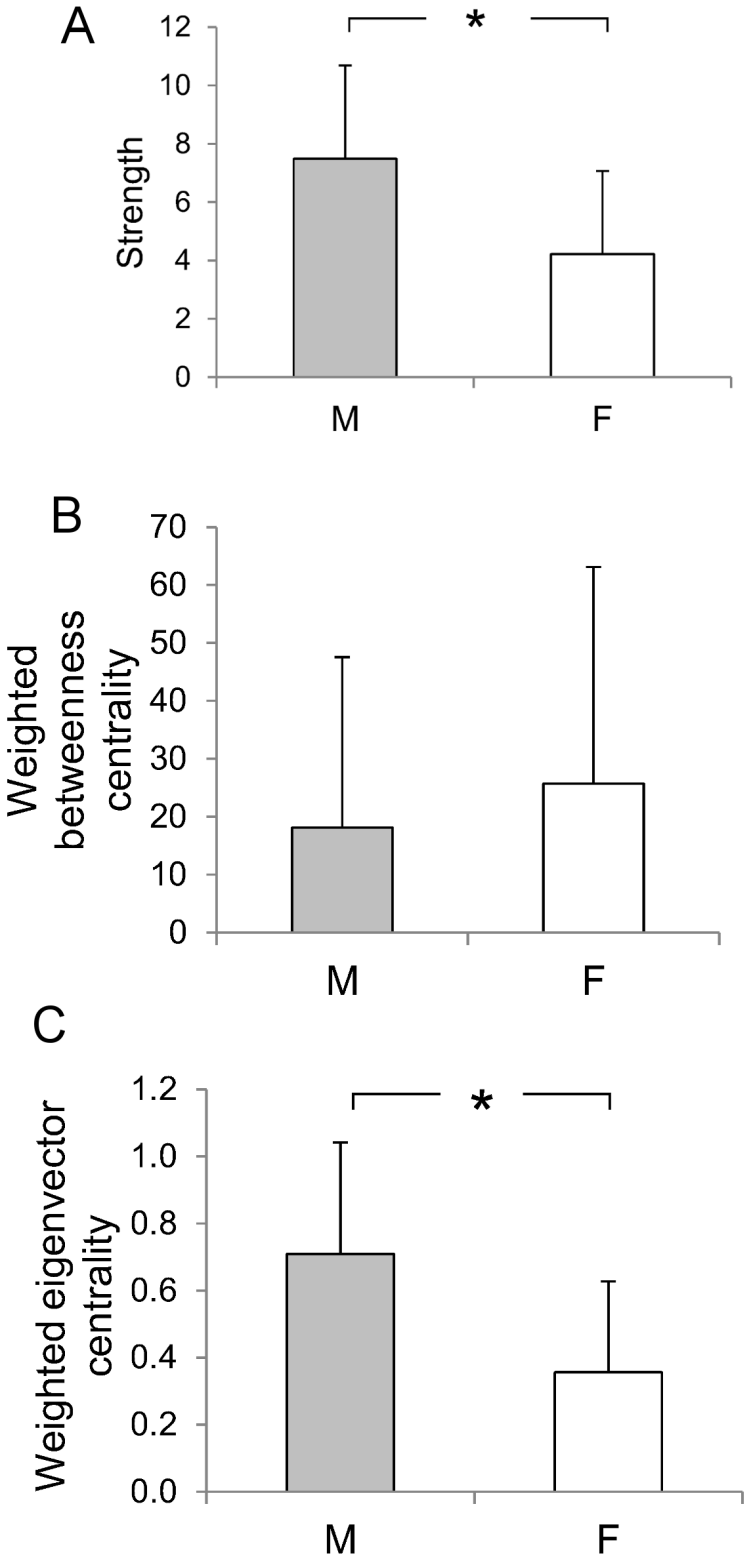
Mean (+SD) of (a) strength, (b) weighted betweenness centrality and (c) weighted eigenvector centrality scores for male (M) and female (F) chimpanzees. Asterisk indicates significant difference after the Bonferroni correction.

The chimpanzee network was very dense (Figure 5), with 1368 of the possible 1485 connections (92%) present. Individuals had an average of 49.7 contacts, almost the entire group, and an average strength of 5.345. The distribution of strength and weighted eigenvector centrality values across individuals were not skewed, while weighted betweenness centrality was positively skewed (Figure 6). Overall, males had significantly higher strength (P<0.001; Figure 7a) and weighted eigenvector centrality (P<0.001, Figure 7c) scores than females but did not differ significantly in weighted betweenness centrality (P = 0.453, Figure 7b).

#### Effect of removals

**Figure 8:**
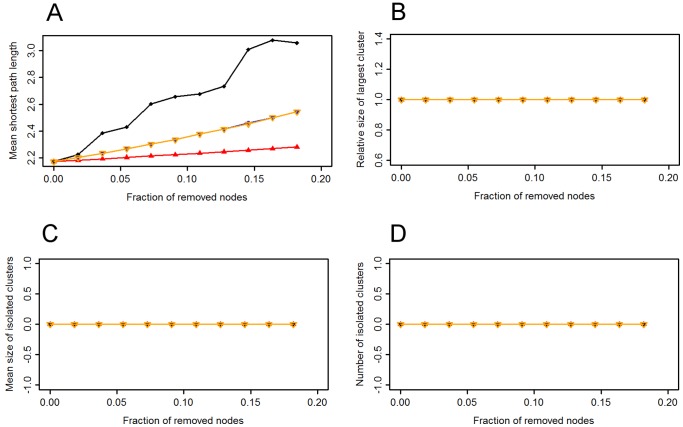
Network metrics plotted against the fraction of removed nodes in the chimpanzee network. The impact of random and targeted removals on (a) the weighted mean shortest path length, (b) the size of the largest cluster (relative to the number of individuals remaining), (c) the mean size of isolated clusters and (d) the number of isolated clusters. Red triangles represent the mean of 10,000 random removals, blue squares targeted removals on individuals with the highest strength, black diamonds individuals with the highest weighted betweenness and yellow inverted triangles individuals with the highest weighted eigenvector centrality.

After the removal of the individuals with the highest strength and weighted eigenvector centrality, the weighted mean shortest path length increased slightly more than under random removals (Figure 8). The weighted mean shortest path length increased more following the removal of individuals with higher weighted betweenness centrality, but the increase was still relatively small. The relative size of the largest cluster did not change following either random or targeted removals; all individuals remained in one cohesive component. Consequently, the mean size of isolated clusters and the number of isolated clusters remained zero.

## Discussion

This study used a social network approach to investigate potential vulnerability to disease and to identify the presence of superspreaders in two species of great apes. Comparisons between these species were limited by the differences in network size and, in particular, the differences in the length of time over which data were collected (orang-utans: 9 years, chimpanzees: 3 years). Although this prevented us from making detailed quantitative comparisons, the markedly different overall patterns that emerged highlight differences in how disease is likely to spread in the two species. The orang-utan network was characterised by sparse and weak connections compared to the density of strong connections in the chimpanzee community, suggesting that disease transmission between individual orang-utans is likely to be limited, in contrast to chimpanzees that are all inter-connected through a range of pathways, allowing for potentially very rapid disease spread. Contagious diseases may thus represent a lesser threat to orang-utans than chimpanzees, which may be a cause of the generally lower mortality seen in this species compared to chimpanzees [41].

It is important to note that this study only focused on within-species disease transmission, while in reality disease often spreads between species [56], including to and from humans [57]–[59]. Furthermore, using networks based on long periods of data collection may lead to overestimating the number of associations present within the community at any point in time. As such, vulnerability to disease may be lower than predicted here (especially in the case of orang-utans). However, the aim of this study was to explore the spread of disease using the ‘general’ social structure of the two species. For this, using weighted networks where edge weights take into account both the frequency of association and observation effort (namely the DAI) should provide a reliable representation of this overall social structure. The orang-utan data used here were from a small number of orang-utans in a relatively limited geographical area [42], covering only a small subset of the entire Sabangau forest population. This is important in relation to migratory individuals and seemingly isolated animals, whose complete range of social relationships may not have been recorded despite the long study period, and as such their role in disease spread may be underestimated. Chimpanzee community membership, on the other hand, is more fixed and so contacts and hence opportunities for disease spread should be more comprehensively and evenly sampled between individuals, although dispersing and immigrating females as well as inter-group encounters will affect disease spread in a way not simulated here. Despite these limitations, our results provide a clear indication of the differences between the species in disease susceptibility and in the importance of superspreaders for potential disease spread.

### Potential Superspreaders

Although most orang-utans had few social relationships, three resident females possessed greater strength and weighted eigenvector centrality than average and two of these females also had considerably greater weighted betweenness centrality than average. These three females thus occupied disproportionately central positions in the network and could potentially therefore become superspreaders in a future disease outbreak, due to the ability to transmit a disease more widely than other individuals. More generally, females and unflanged males had higher centrality than flanged males, suggesting that these two age-sex classes may be more influential in disease spread than the flanged males of this population. At Tanjung Puting, Galdikas [37] found adolescent females to be the most social age-sex class while nulliparous sexually active females and unflanged males were amongst the most gregarious individuals at a number of other sites [39], [60], in line with the findings presented here.

In stark contrast, superspreaders could not be identified in the chimpanzee study community. Although weighted betweenness centrality was positively skewed, strength and weighted eigenvector centrality scores were relatively evenly distributed, with many individuals having high scores. Similarly, at Kibale Forest, Uganda, Rushmore et al. [61] found that the strength distributions in networks (based both on close contacts within 5 metres and party membership) were not skewed towards particular individuals. Nevertheless, our results also show an overall sex difference, with males showing significantly higher centrality scores than females. This is in line with previous studies that have also found males more frequently in parties [62], with a greater tendency to join them [63] spending less time alone [64] and being significantly more gregarious than females [65]. At Kibale, Rushmore et al. [61] found that adult females and juveniles with large families had the highest strength, but also found that high ranking males had high strength in the close contact network. In sum, male chimpanzees are therefore likely to play a more important role in disease spread than females, but the extent of this may vary between sites.

### Comparing Targeted and Random Vaccinations

Orang-utan and chimpanzee networks differed in their susceptibility to fragmentation following removals. Random removals had little impact on the structure of the orang-utan network while targeted removals caused considerable fragmentation. In contrast, the chimpanzee network was only more susceptible to targeted than random removals in one measure, weighted mean shortest path length; however, even this effect was much weaker than that found for the orangutan network. Even after randomly removing 10 individuals (almost 20% of the community), the remaining chimpanzees were connected in one cohesive component.

Greater susceptibility to targeted (but not random) removals has been found in a range of species, such as ground squirrels [20], captive chimpanzees [21], killer whales (*Orcinus orca*) [66], honeybees (*Apis mellifera*) [18] and dolphins (*Tursiops truncatus*) [16], [67], indicating that it may be a common feature of animal societies. However, the extent of this increased susceptibility varies, and this can be used to inform conservation. The results presented here suggest that targeted vaccinations could be an effective preventative measure in orang-utans while random vaccinations are unlikely to prevent or considerably slow the spread of a disease. Targeted vaccinations of potential superspreaders could reduce the number of possible pathways for disease transmission, thus limiting the size and speed of an epidemic. The fact that the orang-utan network fragmented following the removal of specific individuals also suggests that the death of key individuals in this network would have a considerable impact on network connectivity [16]. Thus, the spread of a fatal disease in an orang-utan population is likely to reduce overall cohesion, at least temporarily, which is likely to be very disruptive for a population.

The chimpanzee network, by contrast, was relatively robust against member loss, suggesting that targeting a small number of key individuals for vaccination would not be a very effective method of preventing disease transmission in this species. This is because the high number of links between individuals ensures that disease can spread rapidly. In addition to devastating Ebola outbreaks, a number of chimpanzee communities have been recorded to suffer from respiratory epidemics, many of which were fatal [57], [68]–[70]. As deforestation and human encroachment continue, and chimpanzee ecotourism gains popularity, the risks of inter-specific disease transmission will increase and strategies to cope with disease will be necessary to reduce fatalities. In the case of chimpanzees, vaccination campaigns targeting a small number of specific individuals are unlikely to be very effective, suggesting that other preventative measures, such as the rules and regulations regarding hygiene and maintaining minimal distances from the apes [10], should be given priority.

## Conclusion

The results presented here have implications for great ape conservation strategies. First, they suggest that targeted vaccinations are a potentially valuable preventative measure for orang-utans. Second, although chimpanzees are predicted to be far more susceptible to disease spread than orang-utans, vaccinations of targeted individuals may not provide a useful preventative measure. As there is a severe risk of human diseases spreading to chimpanzees alternative preventive measures need to be prioritised; once disease has penetrated a chimpanzee community, it will be difficult to stop.
